# Training the Immune Response: B-cells' Master Regulator

**DOI:** 10.1371/journal.pbio.0020226

**Published:** 2004-07-13

Viruses, bacteria, and other pathogens betray their presence in the body through exterior proteins, distinct to each strain. To prepare for the multitude of potential infectious agents, developing B-cells shuffle their genes to produce as many as a billion different antibodies, one to match almost any foreign protein. Upon infection, a limited subset of these antibodies will recognize a particular pathogen and mobilize a larger, targeted immune response. B-cells producing the “recognizing” antibody refine and test genetic modifications, adjusting the antibody's fit to the foreign entity. B-cells compete for the best match, or highest affinity; the winners survive to produce more cells and more antibodies against the invader.[Fig pbio-0020226-g001]

**Figure pbio-0020226-g001:**
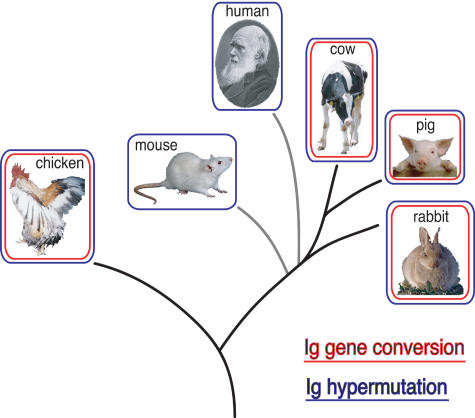
Occurrence of Ig gene conversion and hypermutation on an evolutionary tree

B-cells require an enzyme called activation-induced cytidine deaminase (AID) to develop the most effective antibody. AID generates mutations in the highly variable target-recognition region of an antibody. Removing the AID gene prevents antibody refinement in mature human and mouse B-cells—which use a process called somatic hypermutation to alter single nucleotides in the antibody gene—as well as chicken cells that use a different process called gene conversion to produce variation. Unlike the single nucleotide changes caused by hypermutation, gene conversion modifies an antibody by swapping part of its antigen-binding region for a replacement gene segment. Preference for hypermutation versus gene conversion varies across species, and can even vary within a species. B-cells in chickens use gene conversion through adolescence, when the cells move from a hindgut organ called the bursa into the spleen, where hypermutation takes over.

It is unclear precisely how AID induces either somatic hypermutation or gene conversion, and how it chooses one over the other. Several recent studies suggest that AID's effectiveness may depend on damage to a single DNA base—specifically, changing a cytidine to uracil, which AID can do in either DNA or RNA.

To test whether AID causes hypermutation and gene conversion through a common pathway, Jean-Marie Buerstedde and colleagues at the National Research Center for Environment and Health in Munich, Germany, deleted the donor genes that supply replacement segments for gene conversion in chicken bursa cells. The cells not only stopped performing gene conversion; they revved up single nucleotide mutations in a pattern that looked suspiciously like somatic hypermutation. The mutations targeted hotspots for gene conversion, suggesting that hypermutation and gene conversion share common starting points along antibody genes. This paper adds evidence that AID functions by swapping a single DNA base to induce multiple modes of gene shuffling and refinement in B-cells.

